# Moving from Malaria Burden Reduction toward Elimination: An Evaluation of Mass Drug Administration in Southern Province, Zambia

**DOI:** 10.4269/ajtmh.19-0669

**Published:** 2020-07-02

**Authors:** John M. Miller, Thomas P. Eisele, Maya S. Fraser, Manuel T. Lewis, Laurence Slutsker, Elizabeth Chizema Kawesha

**Affiliations:** 1 PATH Malaria Control and Elimination Partnership in Africa (MACEPA), Lusaka, Zambia;; 2 Department of Tropical Medicine, Center for Applied Malaria Research and Evaluation, Tulane University School of Public Health and Tropical Medicine, New Orleans, Louisiana;; 3 PATH MACEPA, Seattle, Washington;; 4 National Malaria Control Centre, Zambia Ministry of Health, Lusaka, Zambia

## Abstract

From December 2014 to February 2016, a cluster randomized controlled trial was carried out in 60 health facility catchment areas along Lake Kariba in Zambia’s Southern Province. The trial sought to evaluate the impact of four rounds of a mass drug administration (MDA) intervention with dihydroartemisinin–piperaquine (DHAP) or focal MDA with DHAP at the household level compared with a control population that received the standard of care. This study was the first randomized controlled trial with DHAP for MDA in sub-Saharan Africa and was conducted through a collaboration between the National Malaria Elimination Programme in the Zambian Ministry of Health, the PATH Malaria Control and Elimination Partnership in Africa, and the Center for Applied Malaria Research and Evaluation at Tulane University. This article serves as an introduction to a collection of articles designed to explore different aspects of the intervention. By describing the recent history of malaria control in Zambia leading up to the trial—from the scale-up of point-of-care diagnosis and treatment, vector control, and indoor residual spraying early in the twenty-first century, to the efforts made to sustain the gains achieved with that approach—it provides a rationale for the implementation of a trial that has informed a new national strategic plan and solidified malaria elimination as Zambia’s national goal.

## INTRODUCTION

From December 2014 to February 2016, a cluster randomized controlled trial was carried out in 60 health facility catchment areas along Lake Kariba in Zambia’s Southern Province to evaluate the impact of four rounds of a mass treatment intervention with dihydroartemisinin–piperaquine (DHAP). The trial sought to evaluate the relative effectiveness of community-wide mass drug administration (MDA) or focal MDA (fMDA) at the household level (fMDA) with DHAP compared with a control population that received the standard of care (which included good access to case management, including community case management; high coverage of long-lasting insecticide-treated mosquito nets [LLINs] and indoor residual spraying [IRS] with pirimiphos-methyl; and robust surveillance, including rapid reporting and reactive case detection).

This groundbreaking study, the first randomized controlled trial with DHAP for MDA in sub-Saharan Africa, was the result of a collaboration between the National Malaria Control Programme in the Zambian Ministry of Health, the PATH Malaria Control and Elimination Partnership in Africa, and the Center for Applied Malaria Research and Evaluation at Tulane University. Because of the extensive data collection conducted for the trial, many different aspects of the intervention could be addressed in detail. Here, we present a collection of articles designed to explore these topics. This introductory article provides a history of malaria control in Zambia leading up to the trial, a rationale for its implementation, and a brief explanation of the articles found in the supplement.

## ZAMBIA SCALES UP FOR IMPACT

Early in the twenty-first century, Zambia saw its malaria rates tripling over the previous three decades (from 121 per 1,000 population in 1976 to 428 per 1,000 population in 2003).^1^ With the global health and malaria community declaring ambitious malaria targets, the Zambian government identified malaria control as one of its main public health priorities. The country was not without existing prevention measures, but although LLIN distribution channels provided a solid base of coverage, they were disproportionately reaching those in urban areas and those with better access to healthcare facilities, rather than rural populations most affected by malaria.^2^ Thus, with an influx of additional resources planned by the Global Fund to Fight AIDS, Tuberculosis and Malaria and with renewed hope for progress in disease burden reduction, a strategic plan for 2006–2010 was developed emphasizing the scale-up of simplified point-of-care diagnosis with malaria rapid diagnostic tests (RDTs), artemisinin-based combination therapy, vector control with LLINs, and, in selected areas, IRS.^1^ Input from the Bill & Melinda Gates Foundation^3,4^ made mass distributions of LLINs possible, while support from Zambia’s traditional mining centers, the U.S. President’s Malaria Initiative (PMI), and the World Bank led to an increase in IRS coverage by the national program that expanded from the more urbanized rail corridor into more rural malarious districts. In addition, intermittent preventive treatment during pregnancy with sulfadoxine–pyrimethamine for malaria prevention among expectant mothers was supported through antenatal clinics.

With this commitment came noticeable results. More than 6 million LLINs were distributed between 2007 and 2010, and more than 1 million households received IRS annually between 2008 and 2010. Between 2003 and 2010, IRS activities expanded from five districts to 54. By 2010, 73% of households in Zambia had either one or more LLINs or had received IRS in the previous year—a 41% increase in household availability of malaria prevention nationally between 2006 and 2010 and a 5-fold increase between 2001/2002 and 2008.^5^ In the early part of the decade, changes to Zambia’s case management treatment policy, from failing monotherapies to artemether–lumefantrine, were financed to support implementation, although progress in adoption in health facilities was slow.^6^ Between 2003 and 2008, malaria case management was supported by the increased availability of diagnostic tools, primarily through the expansion of RDTs, which were made available initially to rural health centers and health posts. In 2008, more than 2 million RDTs were distributed.^7^

In 2010, Zambia’s malaria focus shifted to sustaining the gains from the initial scale-up. As recommended by a program review in 2010^8^ and adopted in the country’s 2011–2015 strategic plan,^9^ three broad malaria epidemiological strata were defined based on the latest prevalence information from national household surveys. Lusaka and environs were the lowest epidemiological burden, eastern and northern areas of Zambia the highest, and all other areas fell in the middle. Notable new activities during this period included the expansion of malaria testing and treatment services at the community level through integrated community case management (iCCM). The launch of the National Human Resources for Health Strategic Plan in 2011 focused on adding an additional 18,000 health workers in various cadres and 5,000 community health workers (CHWs).^10^ Early research had demonstrated the cost-effectiveness of engaging CHWs to supplement facility testing and treatment services.^11^ Integrated community case management training and funding for implementation ramped up during this period with support from the PMI, the Global Fund, and the Canada International Development Assistance. For the volunteering CHWs offering iCCM services, support was further provided nationally by the roll out of the community health assistant workforce and by increasing the number of health posts in underserved areas.^12,13^ Furthermore, the expansion of malaria case management to more rural, malarious areas through iCCM shifted coverage of care to trained CHWs,^14–16^ addressing the most significant hurdle to diagnosis and treatment access.

In 2011, Zambia began using the District Health Information System, or DHIS2, an open-source health management information system platform that enabled the ministry of health to strengthen their routine health information systems, harmonize reporting across partners, and introduce a weekly malaria rapid reporting system via mobile phone–based data entry. The increasing timeliness and quality of data facilitated the ability to make strategic, data-driven decisions to improve service delivery. In 2011, Zambia initiated CHW-based passive and reactive case detection, expanding care and treatment access to communities and introducing a case investigation intervention to find additional malaria infections in the community.

## SUSTAINING MALARIA CONTROL

Despite often achieving high coverage with vector control and other interventions, by 2014, Zambia had not achieved the reduction in transmission it had expected. Malaria indicators surveys in 2010 and 2012 showed that 16% and 15% of children tested positive by microscopy, respectively, an increase from the 10% documented in 2008.^17–19^ Of note, many of these infections were in asymptomatic individuals likely because of acquired immunity; thus, passive case detection methods seemed unlikely to substantially reduce the parasite reservoir in the population. These challenges to the “control-only” approach and rising global interest in malaria elimination helped move Zambia toward setting an ambitious malaria elimination target date of 2021. To achieve this, new tools were needed and various approaches to population-based drug strategies were considered and tested.

Although MDA had been used in many different countries for malaria control in past decades, it had largely fallen out of favor because of concerns about the transient impact and drug resistance. However, as the global progress began to stagnate between 2000 and 2010, the malaria community began looking for alternative tools. Mass drug administration and related activities—mass test and treat (MTAT), mass screen and treat, and fMDA—began to be re-examined as potential solutions. Mass drug administration had a long, successful history of control and elimination for some neglected tropical diseases, and the advent of artemisinin-based combination therapies meant that resistance was less likely.^20,21^ However, the evidence surrounding these solutions was incomplete, having mostly been generated decades ago using nonexperimental study designs that limited the evidence generated. In addition, many MDA interventions were one-off research exercises that did not include robust malaria prevention and control activities after cessation of the study.^22^ A 2013 Cochrane review concluded that MDA was likely safe but noted the lack of high-quality evidence and called for studies that looked at how the effects of MDA could be sustained past 6 months after administration.^23^ Early modeling results also suggested that MDA could have a significant impact when coupled with other interventions such as robust vector control.^24,25^

## A PIVOT TOWARD ELIMINATION

The decision to launch an MDA trial stemmed from the new focus on elimination and the realization that MTAT, a mass treatment strategy the country had already experimented with, was inadequate to achieve substantial transmission reduction. An MTAT randomized controlled trial conducted in Southern Province, Zambia, from 2011 to 2013 showed a statistically significant effect but one far too small to provide major gains toward elimination.^26^ A similar study conducted in Zanzibar showed no effect.^27^ At the same time, reports from several studies showed that HRP2 RDTs were missing a significant number of low-density infections, meaning that a substantial component of the parasite reservoir would not be detected by a test-and-treat strategy.^28–30^ In this context, therefore, MDA was a promising malaria elimination acceleration strategy to test.

This supplement presents the results of the main trial evaluating four rounds of MDA or fMDA with DHAP, compared with a control of no MDA or fMDA.^31^ We also report the findings of several studies conducted concurrently that investigated various aspects of the trial. These include studies on the acceptability of treatment,^32^ MDA coverage,^33^ DHAP efficacy and adherence to the treatment regimen,^34^ RDT performance,^35^ infection incidence,^36^ parasite genotypes before and after the intervention,^37^ human movement and the relationship between travel history and malaria infection status,^38^ insecticide resistance and parasite infection in *Anopheles funestus* mosquitoes in the study area,^39^ and the cost-effectiveness of adding an MDA or fMDA strategy to the standard-of-care malaria control in Southern Province.^40^ Although specific to the trial and study area, many of the issues explored in these articles are germane to consideration of the potential usefulness of population-based drug strategies in other settings. Moreover, the gains in knowledge from this trial helped usher Zambia into a new era of its malaria efforts, providing key evidence to inform a new national strategic plan and solidifying elimination as the national goal.

## References

[1] Zambia Ministry of Health, 2006 A Road Map for Impact on Malaria in Zambia 2006–2010: A 5-Year Strategic Plan. Lusaka, Zambia: Zambia Ministry of Health.

[2] Agha S Van Rossem R Stallworthy G Kusanthan T, 2007 The impact of a hybrid social marketing intervention on inequities in access, ownership and use of insecticide-treated nets. Malar J 6: 13. 17261185 10.1186/1475-2875-6-13 PMC1794246

[3] Chanda P Masiye F Chitah BM Sipilanyambe N Moonga H Banda P Okorosobo T, 2007 A cost-effectiveness analysis of artemether lumefantrine for treatment of uncomplicated malaria in Zambia. Malar J 6: 21. 17313682 10.1186/1475-2875-6-21 PMC1804270

[4] Steketee RW Sipilanyambe N Chimumbwa J Banda JJ Mohamed A Miller J Basu S Miti SK Campbell CC, 2008 National malaria control and scaling up for impact: the Zambia experience through 2006. Am J Trop Med Hyg 79: 45–52. 18606763

[5] Roll Back Malaria, 2011 Focus on Zambia. Geneva, Switzerland: World Health Organization Progress & Impact Series Country Reports No. 2.

[6] Zurovac D Ndhlovu M Sipilanyambe N Chanda P Hamer DH Simon JL Snow RW, 2007 Paediatric malaria case-management with artemether-lumefantrine in Zambia: a repeat cross-sectional study. Malar J 6: 31. 17367518 10.1186/1475-2875-6-31 PMC1832199

[7] Chizema-Kawesha E Miller JM Steketee RW Mukonka VM Mukuka C Mohamed AD Miti SK Campbell CC, 2010 Scaling up malaria control in Zambia: progress and impact 2005–2008. Am J Trop Med Hyg 83: 480–488. 20810807 10.4269/ajtmh.2010.10-0035 PMC2929038

[8] Zambia Ministry of Health, 2010 Zambia National Malaria Programme Performance Review 2010. Lusaka, Zambia: Ministry of Health.

[9] Zambia Ministry of Health, 2015 National Malaria Control Programme Strategic Plan for FY 2011–2016: Consolidating Malaria Gains for Impact in National Malaria Strategic Plan. Lusaka, Zambia: Zambia Ministry of Health.

[10] Zambia Ministry of Health, 2011 National Human Resources for Health Strategic Plan 2011–2015. Lusaka, Zambia: Ministry of Health.

[11] Chanda P Hamainza B Moonga HB Chalwe V Banda P Pagnoni F, 2011, Relative costs and effectiveness of treating uncomplicated malaria in two rural districts in Zambia: implications for nationwide scale-up of home-based management. Malar J 10: 159. 21651828 10.1186/1475-2875-10-159 PMC3121654

[12] Shelley KD Belete YW Phiri SC Musonda M Kawesha EC Muleya EM Chibawe CP van den Broek JW Vosburg KB, 2016 Implementation of the community health assistant (CHA) cadre in Zambia: a process evaluation to guide future scale-up decisions. J Community Health 41: 398–408. 26547550 10.1007/s10900-015-0110-5

[13] Lusaka Times, 2015 Construction of 650 Health Posts on Course for Completion Next Year. Lusaka, Zambia: Lusaka Times.

[14] Seidenberg PD Hamer DH Iyer H Pilingana P Siazeele K Hamainza B MacLeod WB Yeboah-Antwi K, 2012 Impact of integrated community case management on health-seeking behavior in rural Zambia. Am J Trop Med Hyg 87 (Suppl 5): 105–110. 23136285 10.4269/ajtmh.2012.11-0799 PMC3748509

[15] Larsen DA Winters A Cheelo S Hamainza B Kamuliwo M Miller JM Bridges DJ, 2017 Shifting the burden or expanding access to care? Assessing malaria trends following scale-up of community health worker malaria case management and reactive case detection. Malar J 16: 441. 29096632 10.1186/s12936-017-2088-1 PMC5668974

[16] Strachan C Wharton-Smith A Sinyangwe C Mubiru D Ssekitooleko J Meier J Gbanya M Tibenderana JK Counihan H, 2014 Integrated community case management of malaria, pneumonia and diarrhoea across three African countries: a qualitative study exploring lessons learnt and implications for further scale up. J Glob Health 4: 020404. 25520794 10.7189/jogh.04.020404 PMC4267083

[17] Zambia Ministry of Health, 2010 Zambia National Malaria Indicator Survey 2010. Lusaka, Zambia: Ministry of Health.

[18] Zambia Ministry of Health, 2012 Zambia National Malaria Indicator Survey 2012. Lusaka, Zambia: Ministry of Health.

[19] Zambia Ministry of Health, 2008 Zambia National Malaria Indicator Survey 2008. Lusaka, Zambia: Ministry of Health.

[20] von Seidlein L Dondorp A, 2015 Fighting fire with fire: mass antimalarial drug administrations in an era of antimalarial resistance. Expert Rev Anti Infect Ther 13: 715–730. 25831482 10.1586/14787210.2015.1031744

[21] White NJ, 2016 Does antimalarial mass drug administration increase or decrease the risk of resistance? Lancet Infect Dis 17: e15–e20. 27839929 10.1016/S1473-3099(16)30269-9

[22] von Seidlein L Greenwood BM, 2003 Mass administrations of antimalarial drugs. Trends Parasitol 19: 452–460. 14519583 10.1016/j.pt.2003.08.003

[23] Poirot E Skarbinski J Sinclair D Kachur S Slutsker L Hwang J, 2013 Administration of antimalarial drugs to whole populations. Cochrane Database Syst Rev 12: CD008846. 10.1002/14651858.CD008846.pub2 PMC4468927 24318836

[24] Bretscher MT Griffin JT Ghani AC Okell LC, 2017 Modelling the benefits of long-acting or transmission-blocking drugs for reducing *Plasmodium falciparum* transmission by case management or by mass treatment. Malar J 16: 341. 28814310 10.1186/s12936-017-1988-4 PMC5559805

[25] Brady OJ 2017 Role of mass drug administration in elimination of *Plasmodium falciparum* malaria: a consensus modelling study. Lancet Glob Health 5: e680–e687. 28566213 10.1016/S2214-109X(17)30220-6 PMC5469936

[26] Larsen DA Bennett A Silumbe K Hamainza B Yukich JO Keating J Littrell M Miller JM Steketee RW Eisele TP, 2015 Population-wide malaria testing and treatment with rapid diagnostic tests and artemether-lumefantrine in southern Zambia: a community randomized step-wedge control trial design. Am J Trop Med Hyg 92: 913–921. 25802434 10.4269/ajtmh.14-0347 PMC4426577

[27] Cook J 2019 Mass screening and treatment on the basis of results of a *Plasmodium falciparum*-specific rapid diagnostic test did not reduce malaria incidence in Zanzibar. J Infect Dis 211: 1476–1483. 10.1093/infdis/jiu655 PMC10881232 25429102

[28] Stresman GH 2015 Focal screening to identify the subpatent parasite reservoir in an area of low and heterogeneous transmission in the Kenya highlands. J Infect Dis 212: 1768–1777. 26019285 10.1093/infdis/jiv302

[29] Okell LC Bousema T Griffin JT Ouedraogo AL Ghani AC Drakeley CJ, 2012 Factors determining the occurrence of submicroscopic malaria infections and their relevance for control. Nat Commun 3: 1237. 23212366 10.1038/ncomms2241 PMC3535331

[30] McMorrow ML Aidoo M Kachur SP, 2011 Malaria rapid diagnostic tests in elimination settings—can they find the last parasite? Clin Microbiol Infect 17: 1624–1631. 21910780 10.1111/j.1469-0691.2011.03639.x PMC4821879

[31] Eisele TP 2020 Impact of four rounds of mass drug administration with dihydroartemisinin-piperaquine implemented in Southern Province, Zambia. Am J Trop Med Hyg 103 (Suppl 2): 7–18. 10.4269/ajtmh.19-0659 PMC7416977 32618247

[32] Silumbe K 2020 Assessment of the acceptability of testing and treatment during a mass drug administration trial for malaria in Zambia using mixed methods. Am J Trop Med Hyg 103 (Suppl 2): 28–36. 10.4269/ajtmh.19-0663 PMC7416978 32618242

[33] Finn TP 2020 Treatment coverage estimation for mass drug administration for malaria with dihydroartemisin-piperaquine in Southern Province, Zambia. Am J Trop Med Hyg 103 (Suppl 2): 19–27. 32618251 10.4269/ajtmh.19-0665 PMC7416979

[34] Finn TP 2020 Adherence to mass drug administration with dihydroartemisinin-piperaquine and *Plasmodium falciparum* clearance in Southern Province, Zambia. Am J Trop Med Hyg 103 (Suppl 2): 37–45. 32618267 10.4269/ajtmh.19-0667 PMC7416972

[35] Chishimba S 2020 Prevalence of *Plasmodium falciparum* and non-*falciparum* infections by photo-induced electron transfer–PCR in a longitudinal cohort of individuals enrolled in a mass drug administration trial in Southern Province, Zambia. Am J Trop Med Hyg 103 (Suppl 2): 82–89. 32618252 10.4269/ajtmh.19-0668 PMC7416980

[36] Bennett A 2020 A longitudinal cohort to monitor malaria infection incidence during mass drug administration in Southern Province, Zambia. Am J Trop Med Hyg 103 (Suppl 2): 54–65. 32618245 10.4269/ajtmh.19-0657 PMC7416973

[37] Daniels RF 2020 Evidence for reduced malaria parasite population after application of population-level antimalarial drug strategies in Southern Province, Zambia. Am J Trop Med Hyg 103 (Suppl 2): 66–73. 10.4269/ajtmh.19-0666 PMC7416975 32618255

[38] Porter TR 2020 Recent travel history and *Plasmodium falciparum* malaria infection in a region of heterogenous transmission in Southern Province, Zambia. Am J Trop Med Hyg 103 (Suppl 2): 74–81. 32618250 10.4269/ajtmh.19-0660 PMC7416974

[39] Chanda J 2020 Pyrethroid and carbamate resistance in *Anopheles funestus* giles along Lake Kariba in southern Zambia. Am J Trop Med Hyg 103 (Suppl 2): 90–97. 32618244 10.4269/ajtmh.19-0664 PMC7416976

[40] Yukich JO 2020 Cost-effectiveness of focal mass drug administration and mass drug administration with dihydroartemisinin-piperaquine for malaria prevention in Southern Province, Zambia: results of a community-randomized controlled trial. Am J Trop Med Hyg 103 (Suppl 2): 46–53. 32618249 10.4269/ajtmh.19-0661 PMC7416981

